# A Collaborative Approach for the Development and Application of Machine Learning Solutions for CMR-Based Cardiac Disease Classification

**DOI:** 10.3389/fcvm.2022.829512

**Published:** 2022-03-10

**Authors:** Markus Huellebrand, Matthias Ivantsits, Lennart Tautz, Sebastian Kelle, Anja Hennemuth

**Affiliations:** ^1^Institute of Cardiovascular Computer-Assisted Medicine, Charité—Universitätsmedizin Berlin, Berlin, Germany; ^2^Cardiovascular Research and Development, Fraunhofer MEVIS, Bremen, Germany; ^3^German Centre for Cardiovascular Research (DZHK), Berlin, Germany; ^4^German Heart Center Berlin (DHZB), Berlin, Germany; ^5^Department of Diagnostic and Interventional Radiology and Nuclear Medicine, University Medical Center Hamburg-Eppendorf, Hamburg, Germany

**Keywords:** visual analytics, co-learning, machine learning, CMR, human in the loop (HITL), cardiovascular phenotyping, artificial intelligence, classification

## Abstract

The quality and acceptance of machine learning (ML) approaches in cardiovascular data interpretation depends strongly on model design and training and the interaction with the clinical experts. We hypothesize that a software infrastructure for the training and application of ML models can support the improvement of the model training and provide relevant information for understanding the classification-relevant data features. The presented solution supports an iterative training, evaluation, and exploration of machine-learning-based multimodal data interpretation methods considering cardiac MRI data. Correction, annotation, and exploration of clinical data and interpretation of results are supported through dedicated interactive visual analytics tools. We test the presented concept with two use cases from the ACDC and EMIDEC cardiac MRI image analysis challenges. In both applications, pre-trained 2D U-Nets are used for segmentation, and classifiers are trained for diagnostic tasks using radiomics features of the segmented anatomical structures. The solution was successfully used to identify outliers in automatic segmentation and image acquisition. The targeted curation and addition of expert annotations improved the performance of the machine learning models. Clinical experts were supported in understanding specific anatomical and functional characteristics of the assigned disease classes.

## 1. Introduction

In recent years publications and product developments have shown the potential of artificial intelligence systems in cardiovascular medicine (1–4). Especially data-driven machine learning models can support automatic interpretation of complex spatio-temporal information such as ECG or image data, and the integrated analysis of complementary data from electronic health records, sensor systems, etc. Two factors that are essential for the successful deployment of AI solutions for image-based and multi-modal data interpretation are the model design and training and the interaction with the users (3, 5).

### 1.1. Integration of Image-Based Information in Multi-Modal Cardiac Disease Classification

Integrating complementary data of different types such as demographic information and laboratory and image data requires complex models that filter the densely sampled image information appropriately. Many approaches for phenotyping or predictive modeling using multi-modal data integrate image information *via* conventional clinical parameters such as the stenosis degree or the ejection fraction (6, 7). Thereby valuable feature information of contained in the comprehensive image data might be neglected. In contrast to the traditional features, which describe the heart chamber volumes and myocardial motion patterns of the left and right ventricle, so-called radiomics features describe shape and texture properties of segmented regions context-independently based on image intensities and voxel classification (8). Radiomics features extracted from non-contrast cine MRI have successfully been used to differentiate between patients with myocardial infarction (MINF), dilated cardiomyopathy (DCM), hypertrophic cardiomyopathy (HCM), and an abnormal right ventricle (RV) (9–12). Further approaches used features describing the myocardial texture in MRI-based T1 and T2 maps (13) or delayed enhancement MRI (14) to differentiate myocardial pathologies.

Standard radiomics features, which can be calculated with freely available libraries such as *pyradiomics* were designed for the assessment of compact structures such as tumors (15). To better consider the complex structure of the heart, further features have been suggested. The Minkowski-Bouligand dimensions, as described by Captur et al. (16) assesses how the length or complexity of a contour increases while increasing the scale or detail at which it is measured and is used to assess the trabecularization of myocardium. Further features specifically describing the cardiac anatomy such as the septum thickness have been suggested by Tautz et al. (17, 18).

### 1.2. Expert Annotations for Cardiac Image Interpretation

Quantitative and radiomics analysis of cardiac MRI image data usually requires segmentation of the relevant anatomical structures (19). Recent publications demonstrate the potential of deep learning models such as the U-net for the segmentation and interpretation of typical imaging sequences such as short-axis cine MRI (20). However, the performance of these models depends on the quality of the training data, and previous studies showed that the annotation performance of clinical experts is influenced by the annotation framework (21). The “Society for Cardiovascular Magnetic Resonance” (SCMR) recommends analyzing image frames in end-diastole (ED) and end-systole (ES) (19) for assessing the global cardiac function. Therefore, clinical datasets are often only sparsely annotated, and interactive intelligent annotation and correction tools are required to extend and improve the data so that they can be used to train machine learning models. Commercial medical products might be used if the software offers the export of the expert segmentation in an open format. Open-source application such as 3D Slicer (22), MITK Workbench (23) also provide generic tools for interactive (24, 25), semi- and fully-automated segmentation algorithms. These software tools integrate open-source libraries such as *MONAI Label*^1^ to support an efficient interaction between the annotation and machine learning environment (26). Specialized research software tools such as *Segment* (27) and *CAIPI* (28) provide dedicated solutions for the annotation and processing of four-dimensional cardiac data, which can be used to generate training data. The International Radiomics Platform (IRP) (29) supported by the German Radiological Society^2^ further enables the combination of annotated image data with clinical data and questionnaires.

### 1.3. Clinical Integration of AI-Based Solutions for Cardiac Image Interpretation

Modern deep learning models can classify several cardiac diseases directly from image data (30, 31), but the inference process is hardly understandable for most clinical experts. Explainability approaches for convolutional neural networks support the identification of image regions, which contribute to classification results (32) and provide information for plausibility checks as demonstrated for the interpretation of echocardiograms (33). Explainability methods have been suggested for enhancing the classification of cardiac diseases. Interpretability methods such as *Discovering and Testing with Concept Activation Vectors (D-TCAV)* can be used to show underlying features of the classification (34). Especially in cardiovascular research, it can be highly beneficial for hypothesis generation to understand the shape and tissue characteristics, which determine the assignment of a patient to a particular class. Working with well-defined features, as suggested in Radiomics (8), might enable a compromise between the optimal consideration of the complex image information and a classification that is understandable for clinical experts (35). However, the complex multi-modal data used in phenotyping are difficult to interpret for humans with classical approaches such as heatmaps and two-dimensional diagrams (36, 37). When omics or image data is involved there is a lack of backtracking within these tools, which links the classification to specific relevant locations or time frames of the underlying data.

Integrating AI training setups into clinical environments faces several ethical and legal challenges. The management of health record data is defined by the General Data Protection Regulation (GDPR)^3^. These regulations define how and for what purpose health data can be accessed. Platforms for federated AI training such as JIP (38) and QuantMed (39) provide interfaces for loading data from the Picture archiving and communication system (PACS) and sharing fully trained models in a secure and compliant way. Moreover, JIP implements an interface to connect open-source deep learning libraries and permits the integration of other image processing frameworks like MITK or platforms like IRP (29).

The FDA guidelines address the problem of the need for AI model adaptation and improvement through retraining, and suggest an efficient dynamic process for the development and quality assurance of DL/ML methods in medical image processing (40). The document describes how to manage data, re-train, evaluate, and update AI methods in clinical settings in such a way that newly trained models fulfill the regulatory requirements for a medical product.

### 1.4. Goals

We hypothesize that a dedicated setup for the training and application of machine learning methods with an expert-in-the-loop approach can speed up and improve the training of the AI models for image processing and multi-modal classification. Furthermore, it can support the clinical expert in exploring and understanding the analyzed datasets.

The existing infrastructure and tool solutions presented in the previous paragraphs address all aspects required to set up an environment that supports the development and application of machine learning methods for the integrated usage of cardiac MRI data in multi-modal data classification. Based on these building blocks, we present a concept for a central environment that supports dynamic machine learning with experts in the loop. This central infrastructure should manage data and the training and inference of machine learning models for multi-modal cardiac data interpretation. We envision central modules for cardiac structures segmentation, an automated pre-processing and features extraction process, and a multi-modal cardiovascular disease classification. To integrate clinical experts into the loop we suggest an interactive exploration of the extracted data and a disease hypotheses generation method. Furthermore, this module should provide an interactive data correction and data integrity check, as well as dynamic updates of machine learning models. We test the presented setup with two use-cases and publicly available data from MICCAI challenges on image-based disease classification: the ACDC challenge for the classification of cardiomyopathies (41) and the EMIDEC challenge for the detection pathologies (49) using cardiac MRI and non-image information.

## 2. Materials and Methods

**Figure 1:**
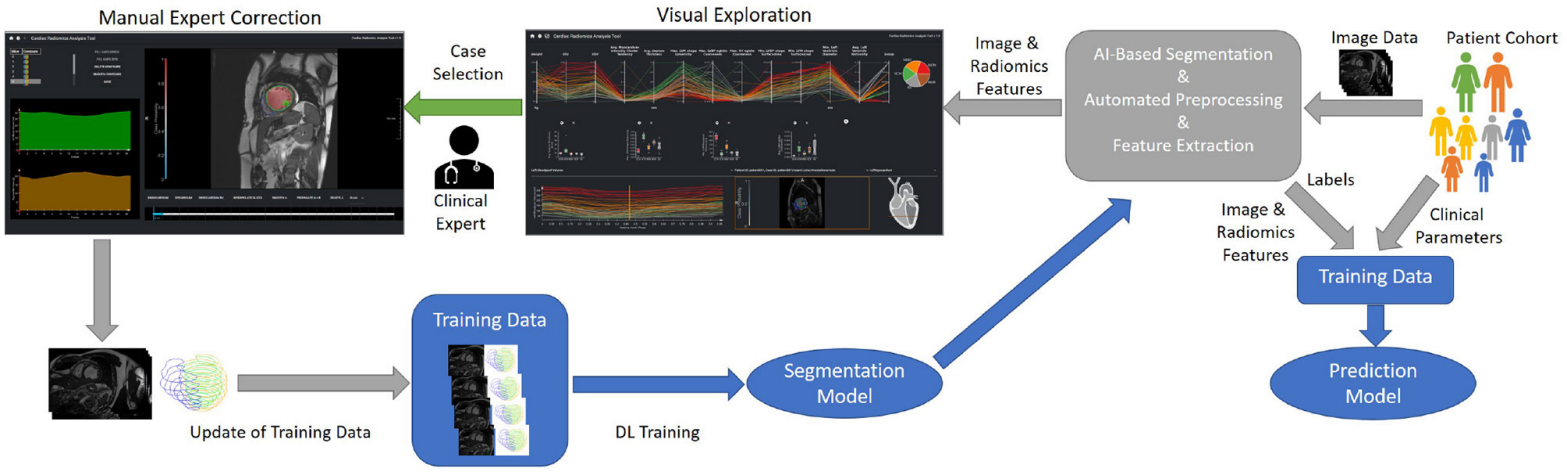
Concept of an iterative process for the training and evaluation of the ML-based segmentation and classification models. On import, image data is automatically segmented, pre-processed, and features are extracted. The results of the automatic segmentation and classification is displayed in the visual exploration interface. In addition clinical experts can manually correct the segmentation results of detected outliers. These corrections can be used to refine the segmentation model.

We propose a modular web-based software environment to support co-learning and comprehensive analysis of cardiovascular imaging data (Figure 1). The architecture of our solution contains the following main components: a data model; a semi-automated tool for efficient labeling; extraction of cardiovascular and radiomics features; visual analytics interface.

For integration into the clinical infrastructure, DICOM network services (42) are used to receive imaging data from PACS systems. On arrival of new data, automated processes import, classify, and, depending on the type of data, automatically pre-process, segment datasets, and extract radiomics features. A web-based application is provided for semi-automated segmentation correction as described in Section Data Correction, Data Integrity, and Dynamic Updates of Machine Learning Models. Cases that the users correct can be directly used to improve the segmentation algorithm by re-training. Figure 1 shows the workflow for refining the segmentation and classification solution. Study data can be analyzed in a web-based visual analytics application Section Interactive Multi-modal Data Exploration with Visual Analytics.

### 2.1. Data Model

**Figure 2:**
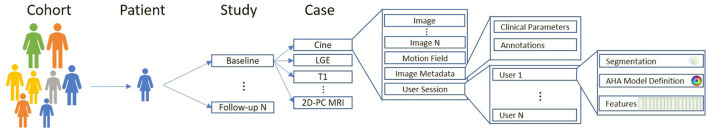
Schematic representation of the data model.

The data model is essential for the traceability of the origin of classification results. Figure 2 shows the major entities and their hierarchical organization. Our data model follows a similar structure described by the DICOM standard (42) using patients and studies as entities to describe a patient cohort. Each patient entity can have one or more studies. Each study can contain several cases containing one or more 3- or 4-dimensional images. For each case, deformation fields, clinical parameters, and annotations, such as image type, or classification labels are stored. Automatic segmentation results and landmarks for the definition the 17-segment model defined by the American Heart Association (AHA) are stored in sessions so that it is always traceable on which data the calculated parameters are based. Furthermore, this data handling allows an evaluation of several readers or repeated measurements and thus supports inter- and intra-observer or ML model comparisons.

### 2.2. Machine Learning for Multi-Modal Disease Classification

#### 2.2.1. Automatic Segmentation, Pre-processing, and Feature Extraction

The image processing part of the application is developed with MeVisLab (43). For data pre-processing and the training of models for the slice-wise segmentation of cardiac structures, we use the Redleaf framework, which allows the integration of inference methods directly in the MeVisLab based applications. U-nets are trained for the segmentation of the relevant structures such as RV, LV, and myocardium (44). These segmentations form the basis for the extraction of typical radiomics features and image-based cardiac biomarkers as suggested in Section Introduction. For 4D image data we generate radiomics feature curves that provide dynamic changes and motion patterns. These time-resolved features are aggregated using minimum, maximum, median, and (arithmetic) mean.

#### 2.2.2. Multi-Modal Cardiovascular Disease Classification

The classifications are based on features describing local and global cardiac function, and radiomics features. We apply eight-fold nested cross-validation to select relevant feature classes. Moreover, during the cross-validation, we perform a model selection of five classifiers and their respective hyperparameters and train a classifier as described in Ivantsits et al. (45).

We perform a feature importance analysis as (46) proposed. This analysis can be performed on any fitted model by calculating a base score produced by the training or test set model. This is followed by a random shuffle to one of the features and compared to the baseline's predictive power. This procedure is then repeated and applied to all features to come up with an importance score. This importance analysis gives insights into the decision made by a classifier and can further be used to discover potential data integrity issues.

### 2.3. Interactive Multi-Modal Data Exploration With Visual Analytics

**Figure 3:**
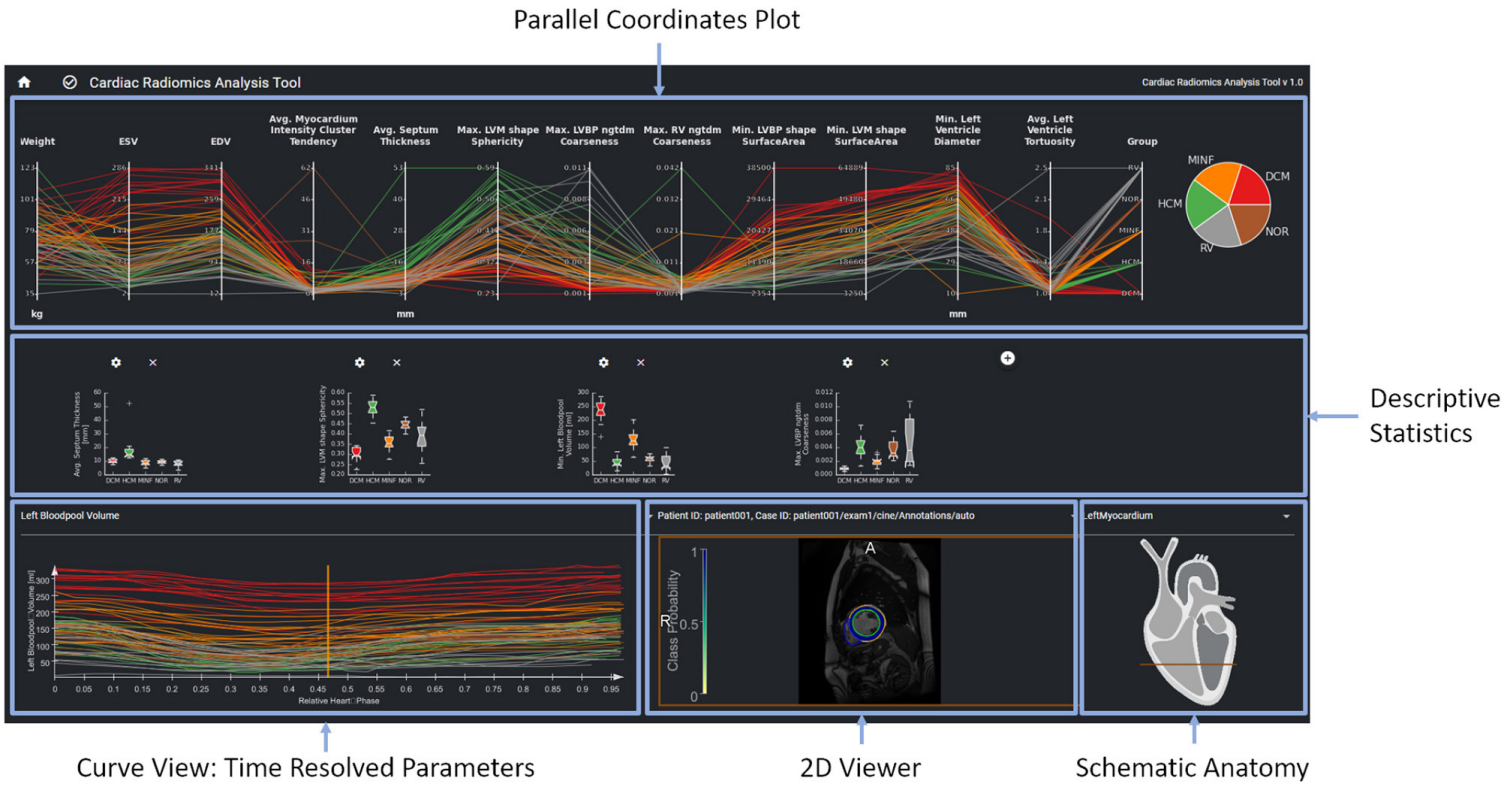
The parallel coordinates plot in the top row shows multi-dimensional data as line sets. Each y-axis represents a specific parameter. The diagrams in the center row show distributions and correlations of selected parameters. The curve view on the bottom left supports the exploration of the temporal dynamics of a selected parameter over the cardiac cycle The image data, the curve parameter of the selected timepoint is based on is displayed in the image viewer on the bottom right. Contours and overlay show the segmentation as well as the local certainty of the segmentation model.

The interactive visualization is designed to support the evaluation, validation, and hypothesis generation. It is provided as a web-based tool for the clinical experts (Figure 3).

The interface provides an overview of relevant features for a given patient cohort. These features are identified by the feature importance analysis of the machine learning module. In order to be sure that features such as, e.g., gender are always considered, users can also select features to be included in the exploration view. The parallel coordinate plots (PCP) visualize the multi-dimensional data as line sets with points representing the datasets' parameters. Each y-axis represents the relevant value range of one parameter. Each line corresponds to one patient dataset. Time-resolved parameters are represented by the aggregated minimum, maximum, or mean values.

Further chart types support an advanced exploration of relationships between different parameters. Scatter plots with regression lines visualize linear relationships between parameters. Histograms show the distribution of different parameters. Box plots give a standardized overview of the data set. Pie charts visualize how frequently individual values or cases of the disease class occur in the study. This can also be used to visually identify unbalanced data sets, for which appropriate measures can be taken in the case of subsequent training.

The exploration tool is designed as a hierarchical tool with different interlinked views. The linking of the data is based on the data model described in Section Data Model. Cases can be selected interactively in the PCP by a technique called brushing (47). The selected parameter range specifies the subset of patients considered for the dependent diagram and curve views. Images can be selected in the 2D viewer from this subset by a drop-down menu above the viewer. The line corresponding to the image selected in the 2D viewer is highlighted in the PCP and the curve diagram by changing thickness and alpha value.

A curve diagram enables the analysis of temporal dynamics of individual parameters. As shown in Figure 3, the curve color corresponds to the class assigned to the underlying dataset to enable a comparison of feature dynamics.

**Figure 4:**
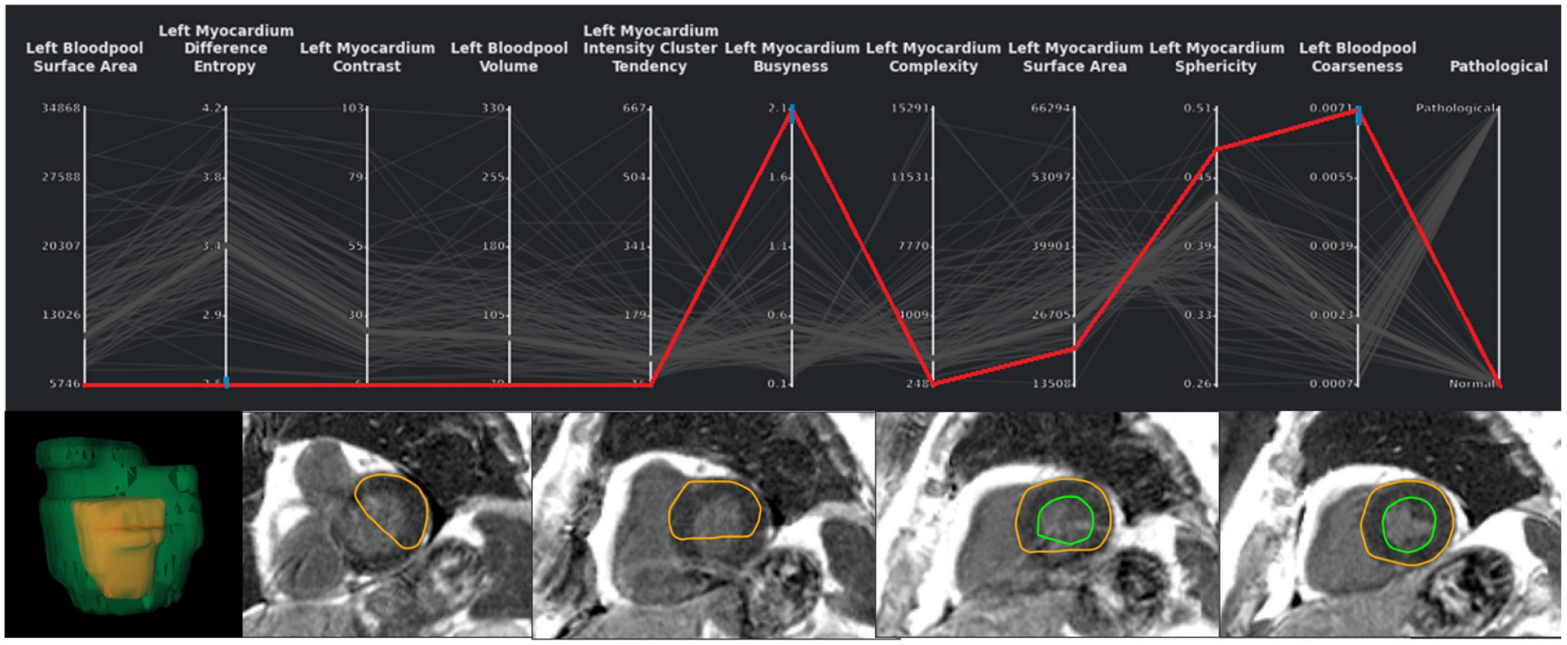
Exemplary case of a segmentation outlier (represented by the red line) in the total EMIDEC study consisting of 100 cases. It can be identified in the PCP by three blue brushes. In the image viewer the erroneous segmentation is clearly visible.

The image viewer shows the image data of a selected case with segmentation contours and an overlay of the segmentation uncertainty. Schematic visualization of the heart shows the position of the displayed image slice with regard to the cardiac anatomy. This approach supports the identification and exploration of outliers as shown in Figure 4 and thereby the data curation. Furthermore, *via* the aggregation of time-resolved data in the PCP the user can backtrack information from a specific feature to the relevant regions within the underlying image. This can further be used to identify any data integrity issues.

#### 2.3.1. Data Correction, Data Integrity, and Dynamic Updates of Machine Learning Models

**Figure 5:**
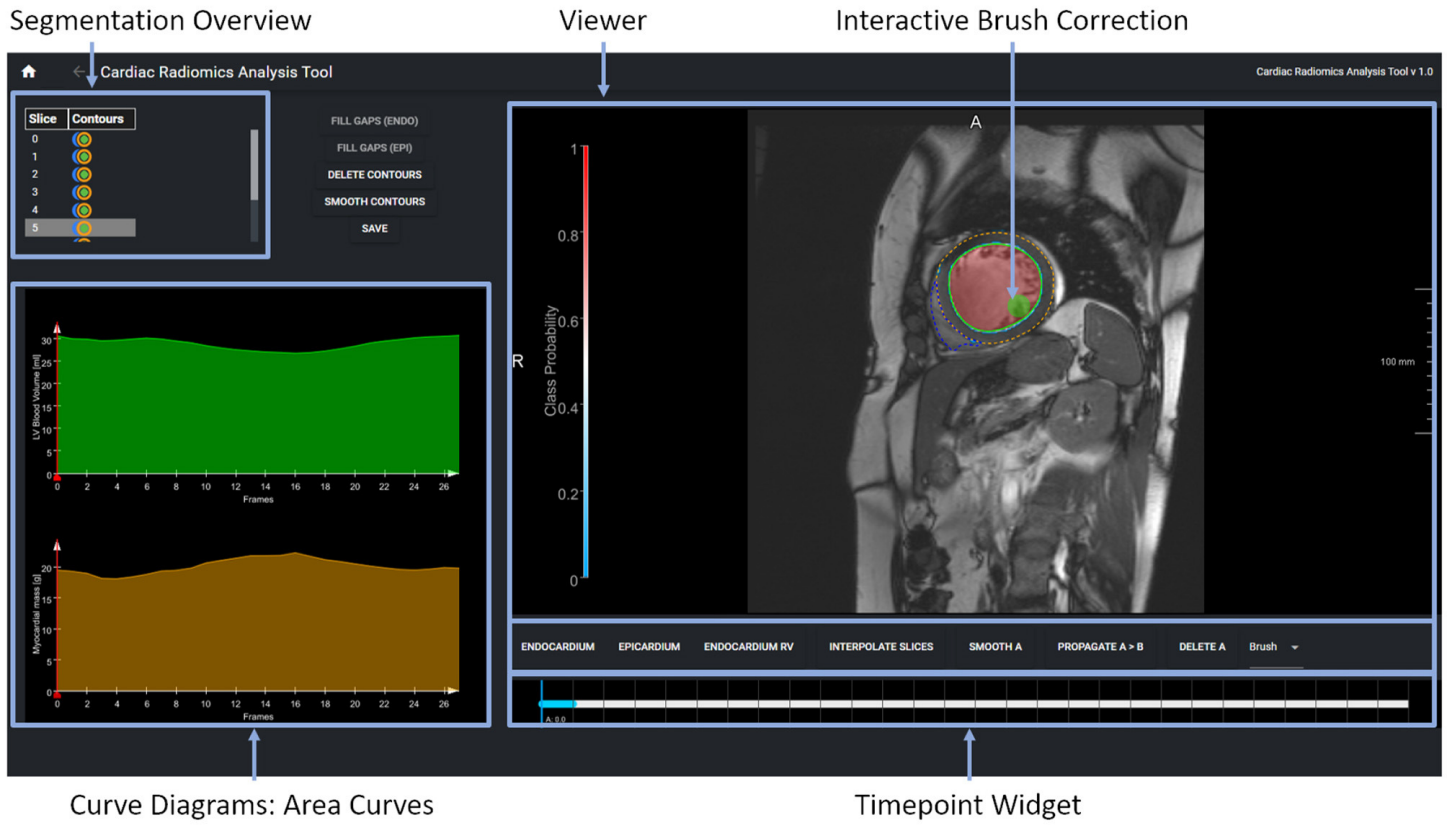
The expert segmentation correction tool shows the segmentation results as contour overlay in the image viewer. The brush tool is visualized as a circle. The table in the upper displays the segmentation status per image slice and anatomical structure. The curve diagrams on the bottom left show the area curves and the segmentation algorithms' probability of the segmentation results on the current slice.

**Figure 6:**
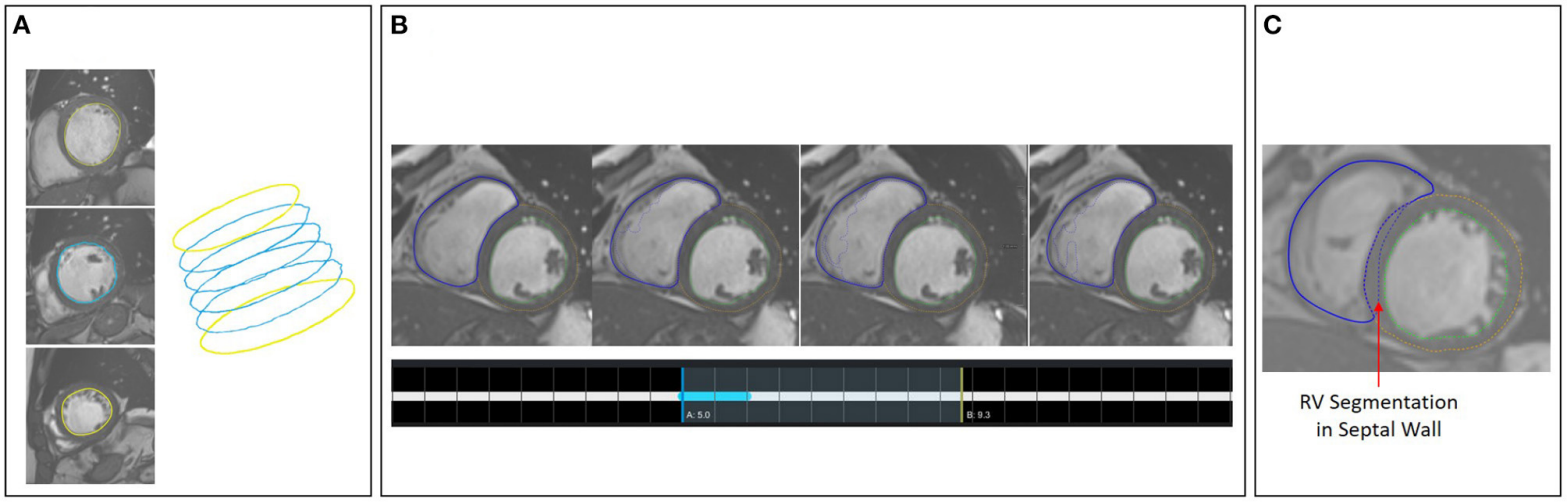
**(A)** Shape-based interpolation: manual contours (yellow) are defined on two slices. The blue contours represent the interpolated results. **(B)** Temporal correction: top row shows a series of time frames of a cine MRI dataset. The solid contour shows the manually corrected contours. The dotted contours represent the incorrect segmentation results. The time-point widgets show the range the user selected for correction *via* contour propagation. **(C)** Correction with logical conditions: the contour of the right endocardium was originally drawn into the septal wall (blue dotted contour). The solid contour represents the corrected segmentation.

A second tool supports the correction and extension of ML-based segmentation results (Figure 5). The image viewer allows to delineate and correct contours defining the anatomical structures (left and right endocardium, left epicardium) using spline, freehand, and brush tools. The overview table indicates the segmentation status of the image slices. When analyzing time-resolved data, such as cine MRI, area and volume as segmentation certainty curve diagrams support the identification of mis-segmented timeframes to reduce the manual interactions. Sparse corrections by the users can be transferred in 3D using shape-based interpolation (25). To transfer segmentation results motion-compensated onto adjacent time frames, we use the deformation field generated by a Morphon-based method (48). In the **timepoint widget** users can specify which timepoints to consider (Figure 6). To help the users to generate consistent segmentation results, an optional tool can enforce that the LV epicardial contour encloses the LV endocardial contour, and that left epicardial and right endocardial contour do not intersect, using spatial set-theoretical boolean operations (Figure 6C). For each individual contour, we store whether it was manually corrected. Thereby, the quality of the automatic segmentation algorithms can be assessed. This information can also identify new cases to improve the AI-based segmentation approach *via* fine-tuning or re-training.

## 3. Results/Application

We test the presented setup with two use-cases and publicly available data from MICCAI challenges on image-based disease classification: the ACDC challenge for the classification of cardiomyopathies (41) and the EMIDEC challenge for the detection of myocardial pathologies (49) using cardiac MRI and non-image information.

### 3.1. Classification of Cardiovascular Disease Based on Cardiac Cine MRI

Cardiac cine MRI provides information on the anatomy and the function of the heart and can help to differentiate between cardiovascular diseases. In this study, we use the freely available dataset from the ACDC challenge (41) to demonstrate how our software environment can be used to support experts in improvement and understanding of cardiomyopathy classification. The dataset comprises normal subjects and patients with one of the following cardiovascular diseases (CVD): previous myocardial infarction (MINF), dilated cardiomyopathy (DCM), hypertrophic cardiomyopathy (HCM), and an abnormal right ventricle (RV). The dataset contains the same number of cases in each subgroup. Clinical experts delineated the left epicardial and endocardial borders in end-systole and end-diastole and assigned the CVD class. The data was acquired on Siemens MRI scanners on 1.5T (Aera) and 3T (Trio Trim); the in-plane resolution was between 1.37 × 1.37 and 1.68 × 1.68*mm*^2^, the slice thickness was between 5 and 8*mm*, distance between slices was 5–10*mm*, and 28–40 phases covered each cardiac cycle. In this study a pre-trained 2D U-Net was retrained on 75 cases from the challenge' training set. The optimal classifier obtained by the described grid search on the ACDC challenge dataset yields a random forest classifier with 230 estimators, the Gini criterion, a maximum depth of six, a minimum of samples per leaf of six, and a minimum sample split of nine. This classifier is built with 112 shape- and texture-based features plus the patients height and weight. For the random forest classifier 80 cases were used for training and 20 cases for testing. **Figure 10A** shows the confusion matrix of this classifier before the correction. This results in an overall accuracy of 0.85, with a precision of 1.0 and recall of 0.75 on RV cases, a precision of 0.75 and recall of 0.75 on normal cases, a precision of 0.67 and recall of 1.0 on MINF cases, a precision of 1.0 and recall of 1.0 on HCM cases, and a precision of 1.0 and recall of 0.75 on DCM cases. Additionally, **Figures 10C,D** illustrate the AUC scores for each individual class plus the macro AUC score of 0.94 before the correction and an AUC score of 0.98 after the correction. After correcting segmentation outliers that were identified *via* the PCP, the accuracy of the classifier improved from 0.85 to 0.9 (**Figure 10B**). Furthermore, **Figure 10E** exemplifies the feature importance of the random forest classifier. We observe the patients' **left myocardium sphericity** to be the most crucial variable in detecting pathological cases, closely followed by the **left blood pool volume** and the **interventricular septum thickness** parameter.

**Figure 7:**
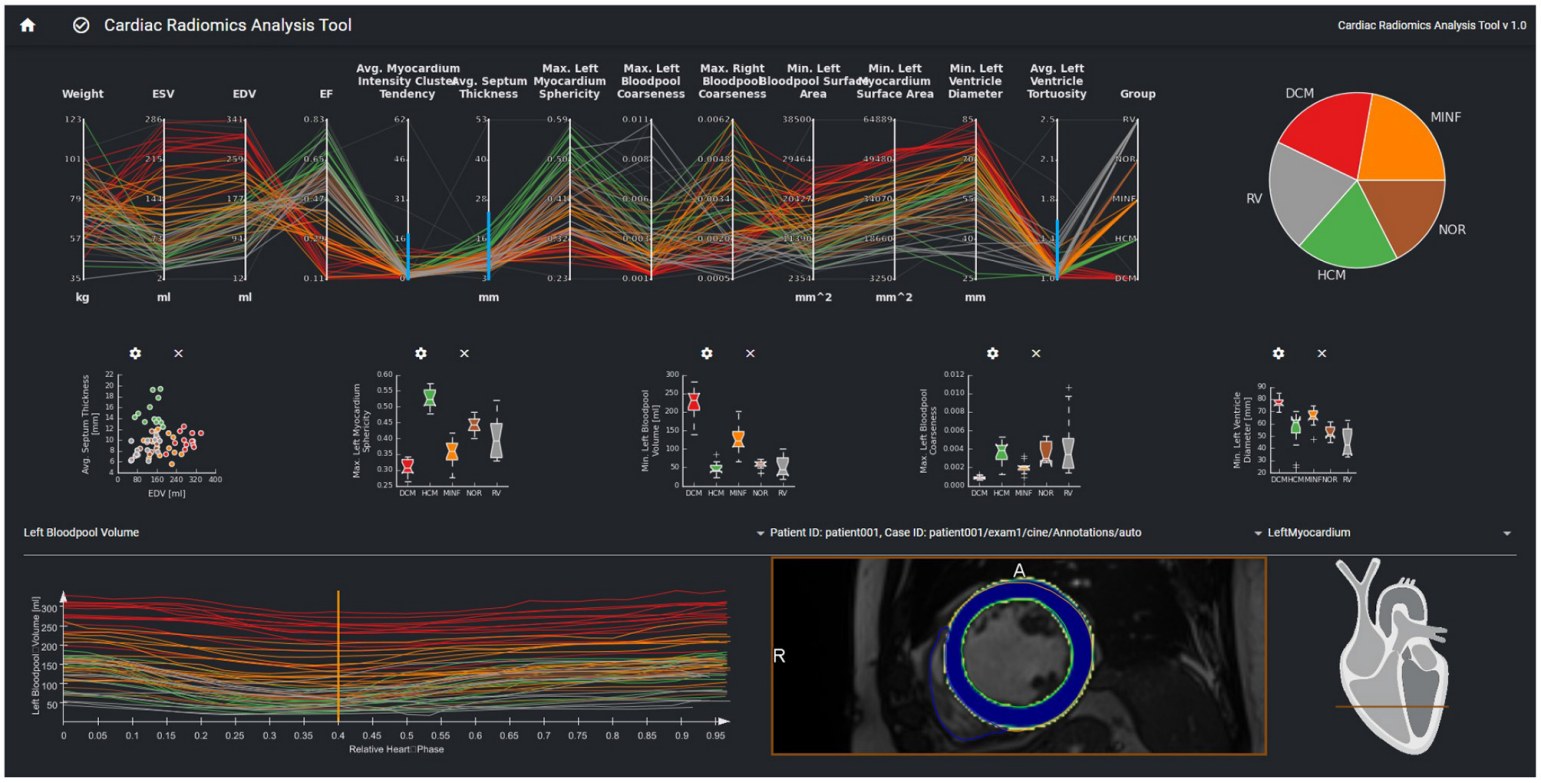
Visual analytics of complete ACDC dataset, which consists of 100 cases. The lines in the PCP represent the patients of the underlying cohort. The color of the lines visualizes the classification result and corresponds to class color in the pie chart. The selection *via* the blue brushes in the PCP includes patients without outliers for features such as septum thickness, myocardium intensity cluster tendency, and left ventricle tortuosity. The scatter plot in the second row shows the EDV vs. avg. myocardium intensity cluster tendency for this cohort. In the box-plots, the distributions of max. myocardial sphericity, max. LV volume and left ventricular diameters in each disease subgroup are shown. The curve diagram shows the LV blood pool curve of each patient over the cardiac cycle.

**Figure 8:**
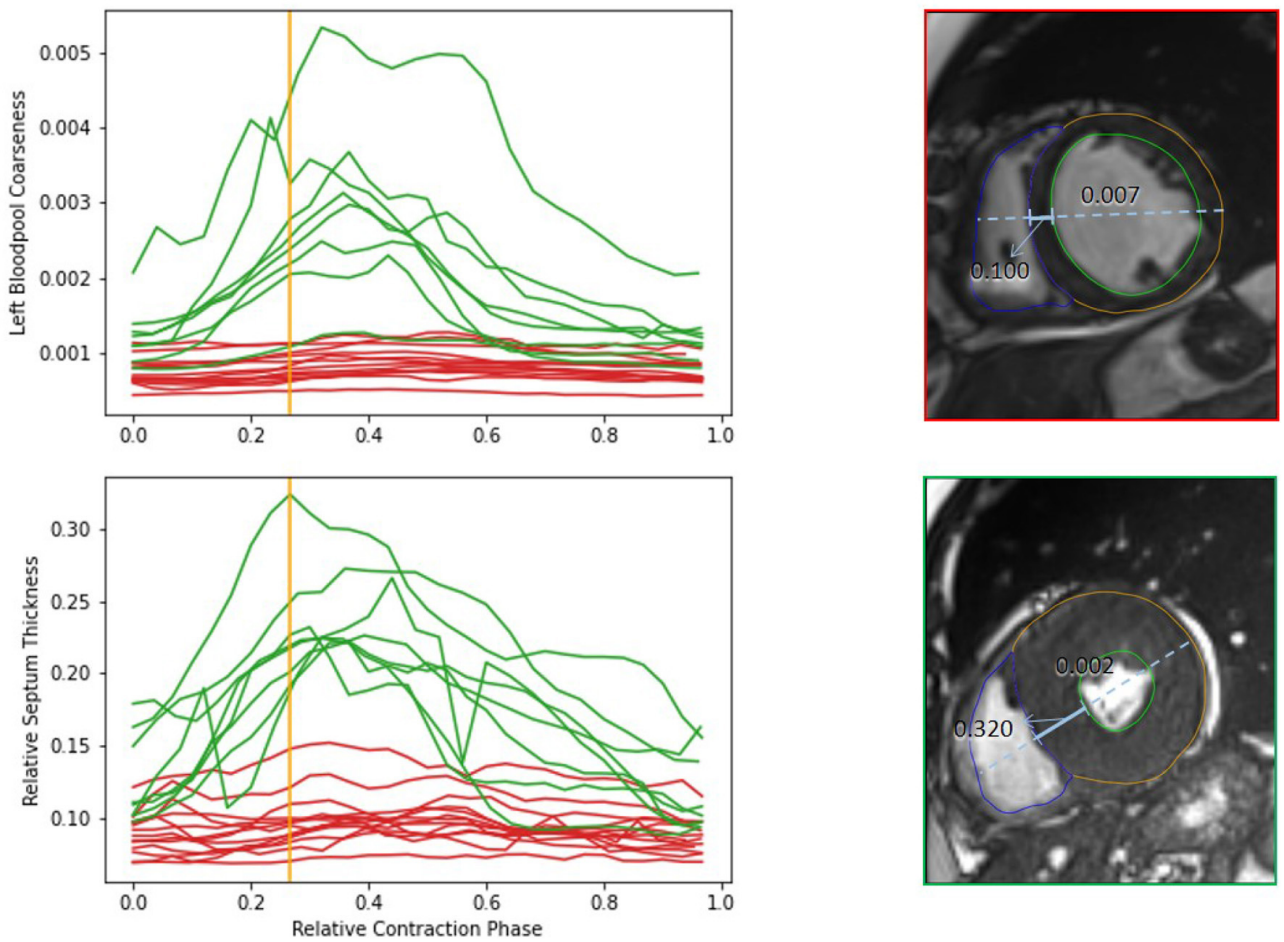
Comparison of features of HCM (green) and DCM (red) patients from the complete dataset. The upper diagram shows dynamics of the left bloodpool coarseness over the cardiac cycle. The lower diagram display the changes of the relative septum thickness. The viewers on the right show the time frames corresponding to the orange line for an example case of a DCM and HCM patient. The solid blue line indicates the septum thickness. The dashed line shows the diameter heart diameter, that is used for normalization of the septum thickness. The coarseness of the selected timepoint is shown on top of the left blood pool. The example cases differ strongly in the anatomical relations as well as in the blood pool intensity distribution.

Figure 7 shows the visual exploration interface for the complete ACDC dataset. Cases of all patients are shown in the PCP. The rightmost y-axis shows the patients' CVD, which is also visualized in the pie chart on the top right. One can see an equal distribution of the diseases in the dataset. Multiple clusters and outliers can be observed. As a first step, clear outliers were removed by deselecting outliers by the average **myocardium intensity cluster tendency**, average **septum thickness**, and average **left ventricle tortuosity**. After removing these outliers, one can differentiate between HCM and DCM patients based on the left blood pool coarseness and relative septum thickness. This can also be seen in the corresponding box plots in the second row. When only selecting patients with HCM and DCM, this becomes even more prominent as shown in Figure 8.

**Figure 9:**
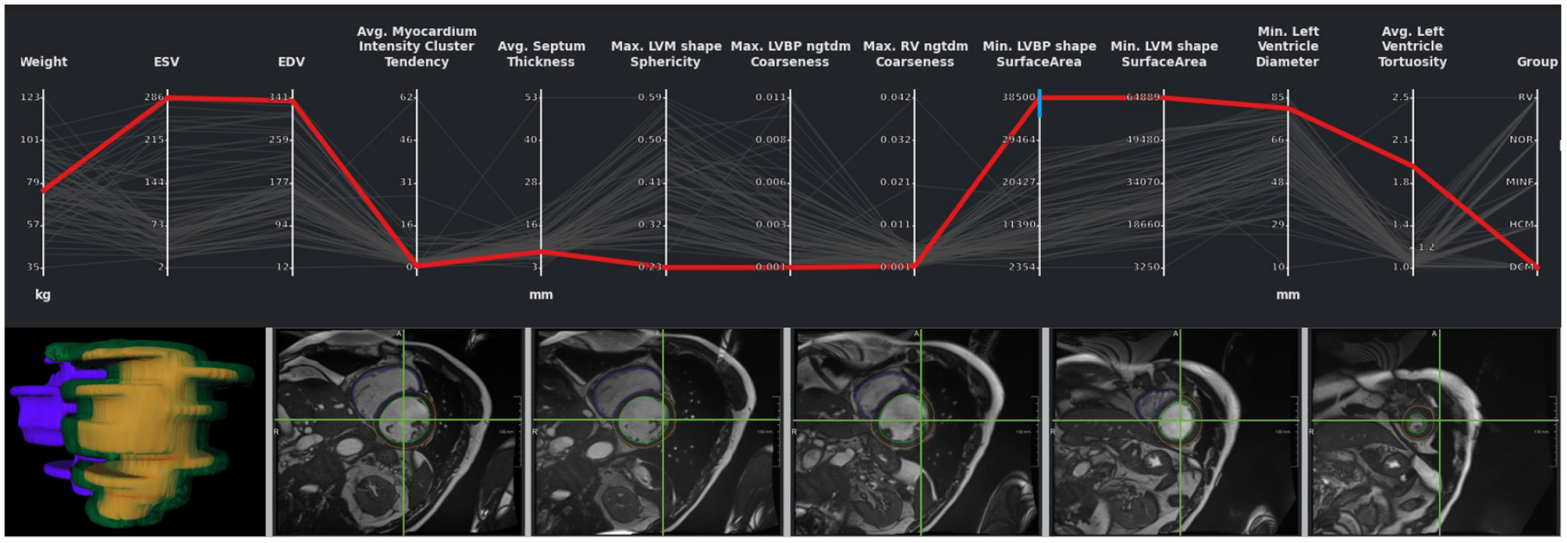
Outlier with motion artifacts. Changes in the left endocardial and epicardial surface area indicate the presence of an outlier. The 3D rendering of the segmentation surface highlights the misaligned slices. The green crosshair in the image viewers was placed in the center of the left blood pool in a basal slice, giving an impression of the motion of the blood pool center.

While outliers were excluded in this first analysis of the study cohort, it is also possible to select and analyze these individual outlier cases. Figure 9 shows cases with strong motion artifacts that could be detected by the left blood pool surface area parameter. The crosshair in the images in the second row shows the center of the left blood pool in the basal slice. The misalignments in the slices can also be depicted in the 3D visualization of the segmentation.

**Figure 10:**
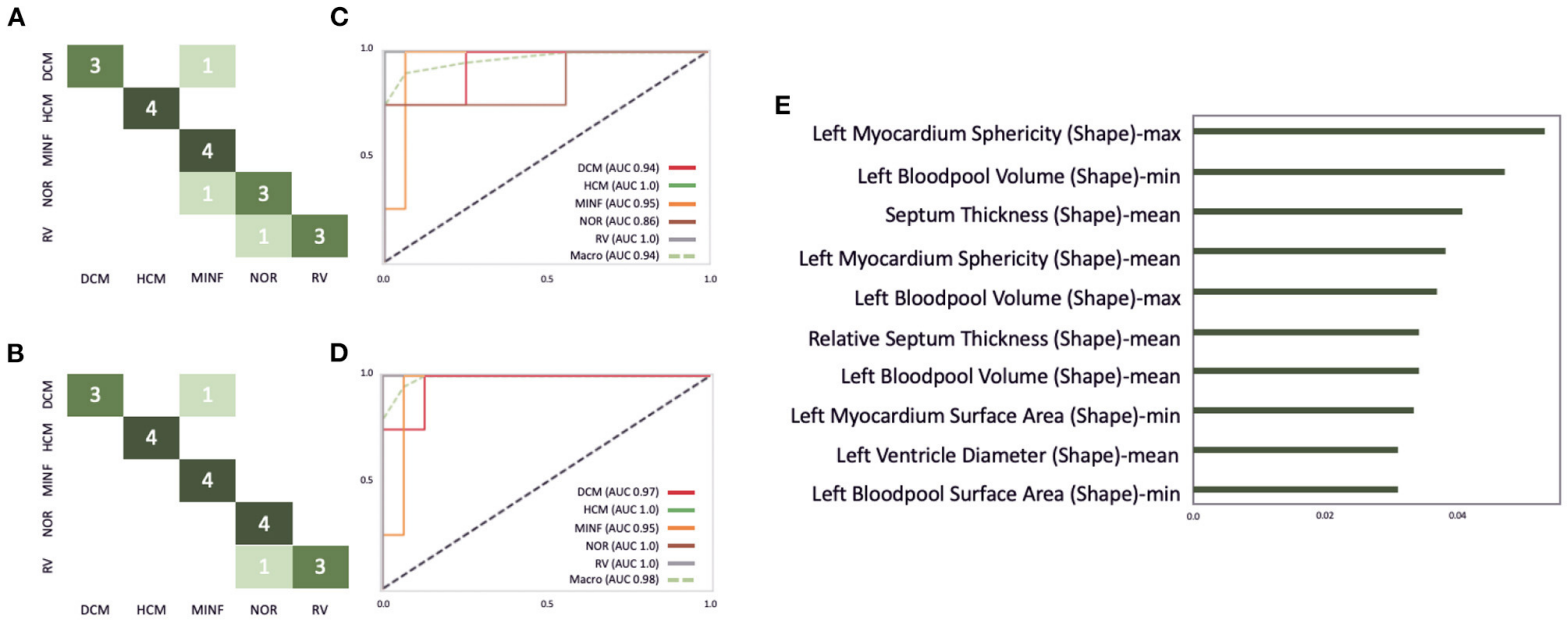
Illustration of the ACDC classifier performance on the test dataset consisting of 20 cases. **(A,B)** Highlights the confusion matrix for the ACDC classifier before and after the correction of image segmentations *via* our described visual outlier detection. Similarly, **(C,D)** show the AUC score improvement of the classifier. **(E)** Depicts the 10 most important features for this classifier.

In outlier cases where the segmentation had failed, it was manually corrected using our labeling interface. After correcting the outlier cases, parameters were extracted again, and the cases were classified again. Figure 10B highlights the classification performance after the correction of the cases as described in Section Interactive Multi-modal Data Exploration with Visual Analytics. We observer an improvement in accuracy from 0.72 to 0.8.

### 3.2. Classification of Normal and Pathological Cases From Late-Gadolinium Enhanced MRI in the Left Myocardium

The EMIDEC challenge (49) provided benchmarking data to assess the performance of segmentation and classification algorithms using clinical parameters and late gadolinium enhancement (LGE) MRI data (50). The dataset consists of 150 cases: 100 diseased patients and 50 normal cases. Patients were split into 100 training and 50 testing sets, containing 1/3 normal and 2/3 pathological cases, which roughly corresponds to real-life observations in the clinical settings. The data was acquired on Siemens MRI scanners on 1.5T (Aera) and 3T (Skyra); the in-plane resolution was 1.25 × 1.25 and 2 × 2*mm*^2^, the slice thickness was 8*mm* and the distance between slices 8–13*mm*. In a post-processing step, the image slices were realigned to prevent any effects of breathing motions.

Analogously to the previous case study, the dataset from the EMIDEC challenge was integrated into our classification and exploration environment. A 2D U-Net was used to generate segmentations of the LV endocardial and epicardial border trained on data from 100 patients with myocarditis and cardiomyopathy to segment the myocardium in the LGE MRI data. As no cases with no-reflow areas were included in the patient data, we also included the 50 unlabeled cases from the challenge's test set. Initial segmentations for these cases were generated by a pre-trained model. An expert used the tools described in Section Data Correction, Data Integrity, and Dynamic Updates of Machine Learning Models to correct these segmentations. Next, the segmentation results were added to the internal cases with expert segmentation, and the final segmentation model was trained. This final model segmented the LV endocardial and epicardial contours and extracted the radiomics parameters on the 80 cases from the challenge's training set. A classifier was trained to differentiate between normal and pathological cases with 25 shape- and texture-based features. We applied a similar strategy as in the ACDC case study. An eight-fold nested CV was used for model and hyperparameters selection. Essential image-based features for the classifiers are shown in the PCP.

**Figure 11:**
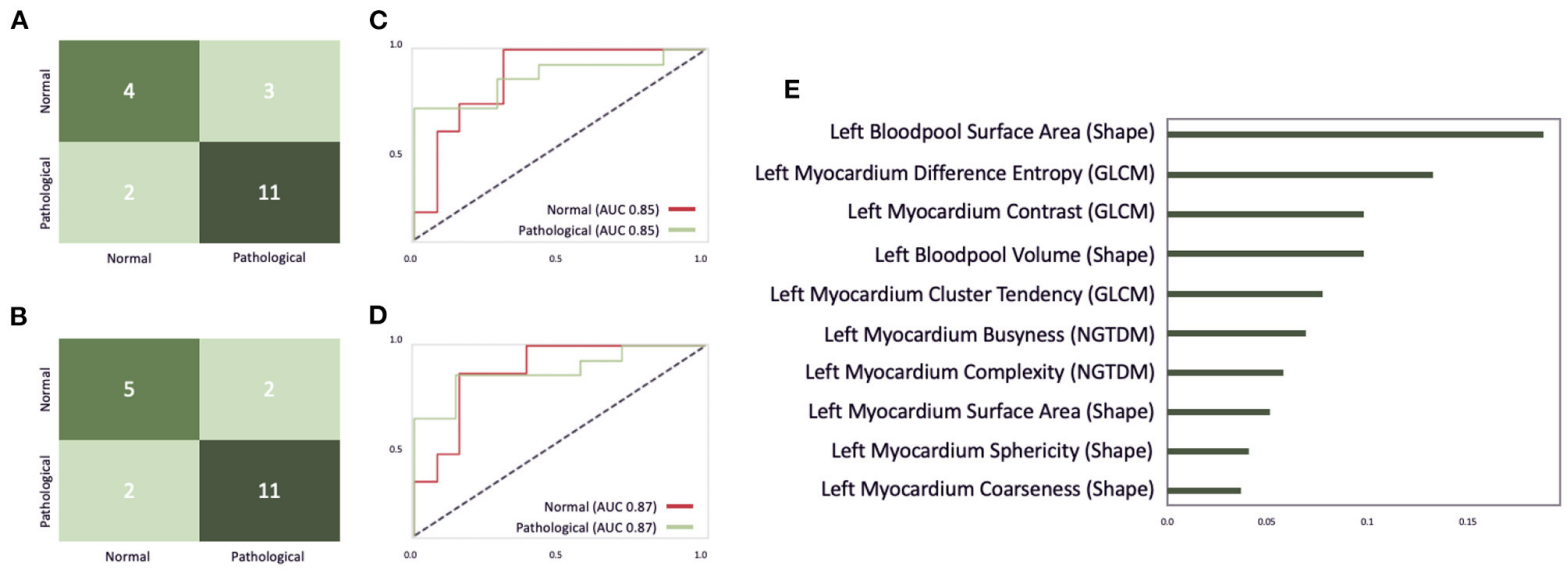
An illustration of the EMIDEC classifier performance on the test dataset consisting of 20 cases. **(A,B)** Show the confusion matrix for the EMIDEC classifier before and after the correction of image segmentations *via* our described visual outlier detection. **(C,D)** Illustrate the AUC score before and after the correction. **(E)** Depicts the importance of the top 10 features for this classifier.

The optimal classifier identified by our grid-search on the EMIDEC challenge dataset turned out to be an extra tree classifier with 190 estimators, the Gini criterion, a maximum depth of six, a minimum of samples per leaf of six, and a minimum sample split of nine. Figure 11A illustrates the confusion matrix of this classifier before the interactive correction. This results in an overall accuracy of 0.75, with a precision of 0.79 and a recall of 0.85 on pathological cases, a precision of 0.67, and a recall of 0.57 on normal cases. Additionally, Figures 11C,D illustrate the AUC scores for each individual class of 0.85 before the correction and an AUC score of 0.87 after the correction. Figure 11E represents the feature importance, where the importance is defined by the difference of the models' baseline and the score after a feature permutation. We observe the patients **left blood pool surface area** to be the most crucial variable in detecting pathological cases, closely followed by **left myocardium difference entropy** and **left myocardium contrast** parameter.

Outliers could be disabled *via* brushing on **myocardial contrast, myocardial cluster tendency, myocardial complexity, left blood pool coarseness** in the PCP analogous to the analysis of the ACDC dataset in Figure 7. After correcting segmentation outliers that were identified *via* the PCP, the accuracy of the classifier improved from 0.75 to 0.8 (Figure 11B).

## 4. Discussion

To test our hypotheses, we applied the presented software environment to two multi-modal machine learning tasks: the classification of patients with cardiomyopathies considering cardiac cine MRI data and the detection of myocardial pathologies considering late gadolinium MRI data. *Via* our visual analytics tools experts could identify erroneous segmentations as outliers in the PCP as illustrated in Figure 10. In both use cases, the performance of the classifier could be improved. The classification accuracy on the test set was improved for pathology detection from 0.75 to 0.8. The accuracy for cardiomyopathies was improved from 0.85 to 0.9. This was achieved by correcting the training dataset and thus the input parameters for the classifier. Consistent with Demirer et al. (21), we found that providing annotation tools with familiar interactions to their routine clinical tools assisted experts with manual corrections. The expert corrections improved the input parameters of single cases, which could then be classified correctly. The suggested visual analytics interface can thus be used to extend approaches, which support retraining models with user-corrected image annotations such as the setup suggested by Dikici et al. (51). Related approaches for the application of visual analytics tools in the exploration of multimodal study data including image information as suggested by Bannach et al. (52) and Angulo et al. (53) strongly focus on the visualization of parameter distributions and have not been applied in a data curation context. However, our solution could also be used for cohort exploration and enhanced by more context-specific visualizations of the cardiac anatomy as suggested e.g., by Meuschke et al. (54). Furthermore, the classification and visualization environment could be used not only for the interpretation of the patient data, but also for a comparison with characteristic cohorts to support the assessment of the certainty and underlying features of the disease classification.

The visualization of the certainty maps produced by the DL-based segmentation, which were displayed as overlays in the image viewers (see Figure 7), did not influence the expert corrections. In future work, we could introduce more sophisticated DL-based outlier detections *via* Bayesian inference as proposed by Gal and Ghahramani (55). This method casts dropout training in DL methods and produces a distribution of outcomes. Unfortunately, this method increases in inference time and can hardly be included in real-time applications. As an additional layer for outlier detection, these certainty maps can be aggregated and displayed in the PCP to give medical experts insights into corrupt results produced by DL models.

The combination of the feature importance analysis and the link to the underlying data model enabled the exploration essential anatomical and functional disease properties in both use cases. Figure 10C illustrates that the most significant parameters for the classification of cardiomyopathies were mostly shape-based parameters. Whereas, the classification of myocardial infarctions was a combination of shape- and texture-based features, as highlighted in Figure 9C. Furthermore, Figure 7 shows that the temporal dynamics of features can also be important for the classification and the understanding of disease types.

Our proposed local and specialized cardiovascular software environment could be successfully applied within a clinical software environment and was used collaboratively by three experts. In order to support multi-centric collaborations, it could be integrated into federated learning platforms such as JIP *via* Docker. This leverages the capabilities of our proposed solution to be applied to federated learning environments that are compliant with GDPR suggestions on health records.

The organization of the training setup follows the suggestions by the FDA (40). However, quality assured model development requires a private validation set to detect model degeneration. This could be added for future applications.

### 4.1. Limitations

The datasets used to test our solution are publicly available and thereby readers can reproduce the described machine learning setup. However, both datasets are relatively small, and the available clinical information is limited. The labels of the EMIDEC dataset are solely based on the inspection of the image data, so that they mean *infarction visible in LGE MRI* and *no infarction visible in LGE MRI* (49). The second label does exclude myocardial pathologies. Therefore the clinical parameters were not included for the interactive optimization of the classifier as described in Section Classification of Normal and Pathological Cases from Late-Gadolinium Enhanced MRI in the Left Myocardium and only integrated for the dataset exploration. Future work with larger datasets will help to further evaluate and improve the presented solution.

## 5. Conclusions and Outlook

We have presented a conceptual design for a software environment that supports the development and application of machine learning methods for multi-modal disease classification using MRI data. We tested the potential of an expert-in-the-loop approach based on visual analysis tools for accelerating algorithm training and for making the learned features understandable with promising results. In future work, we will further quantify the potential of our solution for improving the usage of multi-modal imaging and proteomics data. In addition, we plan to add the monitoring module for an FDA-compliant training setup to offer quality-assured AI solutions. Further clinical studies will have to assess whether an improved disease classification achieved through our setup will have and impact patient outcomes through improved treatment personalization.

## Data Availability Statement

Publicly available datasets were analyzed in this study. This data can be found at: ACDC: https://acdc.creatis.insa-lyon.fr/description/databases.html and EMIDEC: http://emidec.com/dataset.

## Ethics Statement

Ethical review and approval was not required for the study on human participants in accordance with the local legislation and institutional requirements. Written informed consent for participation was not required for this study in accordance with the national legislation and the institutional requirements.

## Author Contributions

AH and SK: funding acquisition. MH, MI, and LT: basic software development and user interface concept. MI and MH: machine learning implementation and validation of machine learning methods. MH, MI, and AH: writing—original draft. All authors helped in conceptualization and writing—review and editing. All authors listed have made a substantial, direct, and intellectual contribution to the work and approved it for publication.

## Funding

This work was partially funded by the BMBF project Berlin Institute for the Foundations of Learning and Data (Grant Number 01IS18037E) and by the Deutsche Forschungsgemeinschaft (DFG, German Research Foundation) – SFB-1470 – B06.

## Conflict of Interest

The authors declare that the research was conducted in the absence of any commercial or financial relationships that could be construed as a potential conflict of interest.

## Publisher's Note

All claims expressed in this article are solely those of the authors and do not necessarily represent those of their affiliated organizations, or those of the publisher, the editors and the reviewers. Any product that may be evaluated in this article, or claim that may be made by its manufacturer, is not guaranteed or endorsed by the publisher.

## Footnotes

^1^Medical Open Network Artificial Intelligence. Available online at: https://monai.io

^2^ https://www.drg.de/de-DE/3601/radiomics/

^3^ https://www.eu-patient.eu/globalassets/policy/data-protection/data-protection-guide-for-patients-organisations.pdf
